# Microglial *Cx3cr1* knockout reduces prion disease incubation time in mice

**DOI:** 10.1186/1471-2202-15-44

**Published:** 2014-03-21

**Authors:** Julia Grizenkova, Shaheen Akhtar, Sebastian Brandner, John Collinge, Sarah E Lloyd

**Affiliations:** 1 MRC Prion Unit and Department of Neurodegenerative Disease, UCL Institute of Neurology, Queen Square, London WC1N 3BG, UK

**Keywords:** Prion disease, Incubation time, Cx3cr1, Microglia, Cytokines, Chemokines

## Abstract

**Background:**

Microglia are resident mononuclear phagocytes of the brain that become activated in response to insults including neurodegenerative diseases such as Alzheimer’s disease, Parkinson’s disease and prion disease. In the central nervous system the chemokine Cx3cl1 (Fractalkine) is expressed by neurons and its exclusive receptor Cx3cr1 is expressed solely on microglia. Cx3cl1/Cx3cr1 signalling is thought to maintain microglia in their resting state and disrupting this equilibrium may allow microglia to become activated. In prion disease, microglial proliferation has been suggested to contribute to overall disease progression, however, in different mouse models of neurodegeneration, loss of Cx3cr1 has been shown to either worsen or improve the phenotype depending on the paradigm.

**Results:**

To investigate the role of Cx3cl1/Cx3cr1 signalling in prion disease we infected *Cx3cr1* null mice with three different strains of prions. Following challenge with Chandler/RML, ME7 and MRC2 prion strains, *Cx3cr1* knockout mice showed highly significant reductions in incubation time. No differences were seen in the pattern and localisation of activated microglia in the brain or in the mRNA expression levels of chemokines/cytokines (*Cxcl10*, *Il-12b*, *Il-1b*, *Arg-1* and *Cxc3l1*).

**Conclusion:**

Our data suggest a protective role for Cx3cl1/Cx3cr1 cross-talk in prion disease.

## Background

Neuroinflammation within the brain is a common feature of neurodegeneration and one of the hallmarks of these changes is the activation of microglia. Microglia are CNS (central nervous system) mononuclear phagocytes that under normal conditions are surveillant but are activated in response to various brain insults [1,2]. Activation can result in phagocytosis and the release of substances such as neurotrophic factors and cytokines/chemokines [3]. While some of these functions are likely to be beneficial, it has been proposed that an excess of particularly proinflammatory cytokines may be damaging and that this immune response may contribute to disease pathogenesis.

Neurons express the chemokine Cx3cl1 (fractalkine) in a membrane bound or soluble form. Its receptor within the CNS, Cx3cr1, is expressed solely on microglia [4-6]. Cx3cl1 is thought to act as a chemoattractant recruiting microglia to sites of injury and the normal signalling between Cx3cl1 and Cx3cr1 is thought to provide part of the neuronal-microglial cross talk that maintain microglia in their resting state [2].

The role of Cx3cr1 has been extensively studied in many models of neurodegeneration and brain injury through the use of Cx3cr1 knockout mice, although often with conflicting results. In a triple transgenic (3xTg: *PS1*_M146V_ knock-in, transgenic *APP*_*Swe*_ and *tau*_P301L_) model of Alzheimer’s disease (AD) it was shown that loss of Cx3cr1 resulted in the increase of microglial migration and prevented neuronal loss while levels of Aβ deposition remained unaltered [7]. The effect of Cx3cl1/Cx3cr1 signalling seen in the 3xTg model contrasts with the findings reported for both the APPPS1 and R1.40 models of AD where loss of Cx3cr1 altered microglial activation and reduced Aβ deposition [8]. Knockout of *Cx3cr1* in a toxin-induced model of Parkinson’s disease (PD) and a genetic model of Amyotrophic Lateral Sclerosis (ALS) (Tg *SOD1*^G93A^) worsened the phenotype, in both cases producing more neuronal loss and, for the ALS model, accelerated deterioration of hind limb grip function and reduced survival were also seen [9]. In related experiments with a toxin-induced model of PD in rats, treatment with the ligand Cx3cl1 was neuroprotective with reduced microglial activation [10]. In addition, in a model of tauopathy, a humanised *MAPT* transgenic mouse also lacking Cx3cr1 showed altered microglial activation, enhanced tau phosphorylation and aggregation as well as poorer spatial working memory [11]. In contrast, smaller infarcts and better functional recovery was seen in the absence of Cx3cr1 in a model of ischaemia [12].

Prion diseases or transmissible spongiform encephalopathies (TSEs) are progressive neurodegenerative diseases that include Creuzfeldt-Jakob disease (CJD) in humans, bovine spongiform encephalopathy (BSE) in cattle and scrapie in sheep [13]. In common with many other neurodegenerative diseases, a key feature of prion diseases is the accumulation of aggregates of an abnormally folded protein. In prion diseases these misfolded protein aggregates propagate by the conversion of normal cellular prion protein (PrP^C^) to abnormal isoforms, designated PrP^Sc^ and constitute lethal infectious agents. Prion infection is accompanied by spongiform change and neuronal loss in the brain. In contrast to other infectious diseases little if any evidence of inflammation is seen in the periphery. However, even before significant neuronal loss occurs, there is widespread activation of microglia, glia and astrocytes in the brain [14-16]. Proinflammatory cytokines increase during disease progression, however, this is greatly reduced relative to that seen during bacterial or viral infections [15,17].

In wild type mice infected with a mouse-adapted scrapie prion strain (ME7) Cx3cl1 is reported to be upregulated in astrocytes but there is no change in surviving neurons and Cx3cr1 expression is also upregulated on microglia [4]. Analysis of hamster brains infected with the prion strain 263K showed that Cx3cl1 is downregulated during disease progression [18]. These data, together with the prominence of activated microglia early in prion disease and the parallels with other neurodegenerative disorders, suggest that Cx3cl1/Cx3cr1 signalling may play an important role in prion disease pathogenesis. To evaluate this hypothesis we challenged Cx3cr1 knockout mice with three different prion strains [5]. Our results show that for two mouse-adapted scrapie strains (Chandler/RML and ME7) and a mouse-passaged BSE strain (MRC2), Cx3cr1 deficiency shortens the incubation time thereby suggesting that Cx3cl1/Cx3cr1 signalling is partially protective in prion disease.

## Methods

### Animals

*Cx3cr1*^*+/-*^ mice (Cx3cr1/GFP, C.129P2-Cx3cr1^tm1Litt^/leg) on a Balb/c background were obtained as embryos from the European Mouse Mutant Archive, resurrected and bred to homozygosity [5]. Wild type Balb/cOlaHsd mice were purchased from Harlan, UK Ltd (Bicester, UK).

### Prion transmission

Inocula for three different prion strains were made from the brains of terminally sick mice as 1% (weight/volume) homogenates in sterile D-PBS. Chandler/RML (I9900) and ME7 (I9459) are two different mouse-adapted strains of scrapie and MRC2 (I9468) is a mouse-passaged strain of BSE [19,20]. Following anaesthesia with isofluorane/O_2_, female mice were inoculated intra-cerebrally with 30 μl of inocula into the right parietal lobe as previously described [21]. Mice were examined daily for clinical signs of prion disease and were culled once a definitive diagnosis had been made or earlier if showing any signs of distress or excessive weight loss. Diagnostic signs of clinical prion disease include ataxia, impaired righting reflex and a sustained hunched posture [22]. Incubation time was defined as the number of days from inoculation to confirmed diagnosis. The Kaplan-Meier log-rank test was used to analyse survival data using the statistical package SPSS (IBM). Animals were housed, maintained and cared for in accordance with institutional, UK and international regulations and standards on animal welfare and conform to ARRIVE guidelines [23]. Ethical approval was granted by the Medical Research Council Prion Unit animal research ethics committee and carried out under UK Home Office licence PPL70/7274.

### Neuropathology and immunohistochemistry

Mouse brains were fixed in 10% buffered formal saline (BFS) and prion-infected tissue was treated in 98% formic acid for one hour to remove infectivity. Tissues were paraffin wax embedded, sagitally sectioned and stained as previously described [22]. Haematoxylin and eosin (H&E) stained sections were assessed to determine the relative levels and distribution of spongiosis and neuronal loss. Prion deposition was evaluated with the anti-PrP monoclonal antibody ICSM35 (D-Gen Ltd, UK) and gliosis was determined with an anti-glial fibrillary acid protein (GFAP) antibody (Dako Ltd, UK). Microglia were visualised by staining with an anti-Iba1 polyclonal antibody (Wako).

### Real-time RT-PCR

RNA was extracted using a TRIzol Plus RNA Purification kit (Life Technologies) from half brains taken from terminally sick prion infected mice and uninfected controls (n = 4 or 5 per group). cDNA was generated using a QuantiTect Reverse Transcription Kit (Qiagen). Contaminating genomic DNA was removed during the extraction with a DNase I digestion and this was confirmed by including a no reverse-transcription control for each sample. Real-time RT-PCR reactions were carried out using Taqman Gene Expression Master Mix enzyme (Life Technologies) on a 7500 Fast Real-time PCR System (Life Technologies). Gene specific Taqman Gene Expression assays (Life Technologies) were used for *Cxcl10*, *Il-12b*, *Il-1b*, *Arg-1* and *Cx3cl1*. Each reaction was duplexed independently with two endogenous controls (rodent *GAPDH* and mouse *β-actin*, Life Technologies). All reactions were carried out in triplicate. Statistical tests were carried out using GraphPad InStat (GraphPad Software, Inc, California, USA).

## Results

### Transmission of prions to *Cx3cr1*^*-/-*^ mice

**Figure 1:**
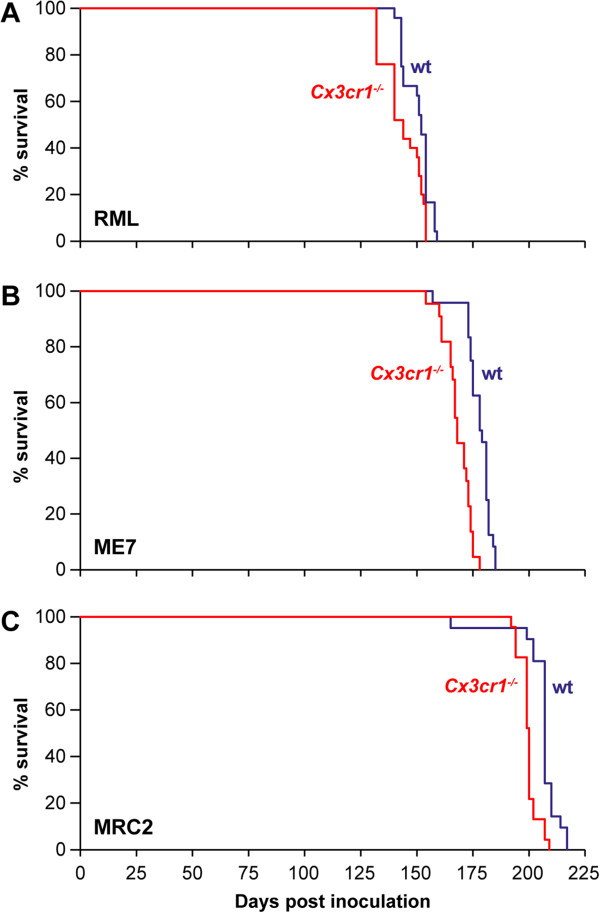
**Kaplan-Meier survival curves.** Data are shown as % of surviving animals (y-axis) plotted against the number of days post-inoculation (x-axis). **(A)** Transmission of Chandler/RML prion strain to *Cx3cr1*^*-/-*^ (n = 25) and *Cx3cr1*^*+/+*^ (WT, n = 24) controls **(B)** Transmission of ME7 prion strain to *Cx3cr1*^*-/-*^ (n = 22) and *Cx3cr1*^*+/+*^ (WT, n = 24). **(C)** Transmission of MRC2 mouse adapted BSE prion strain to *Cx3cr1*^*-/-*^ (n = 23) and *Cx3cr1*^*+/+*^ (WT, n = 21). A reduction in mean incubation time of 4%, 7%, and 7% was seen in **A**-**C** respectively. This reduction in survival was statistically significant for each transmission P = 0.004 **(A)** and P < 0.0001 **(B and C)**, Kaplan-Meier log-rank survival test.

*Cx3cr1* knockout mice were generated by replacing the *Cx3cr1* gene with the green fluorescent protein (GFP) reporter gene [5]. The gene targeting results in the loss of the wild type *Cx3cr1* mRNA transcript and the generation of a chimeric transcript of an untranslated *Cx3cr1* exon spliced to the *GFP* exon. The mice have a normal lifespan with no visible abnormal phenotype. The presence of GFP has not been reported to interfere with microglial function and microglia are still able to become activated in the absence of Cx3cr1 [5]. To investigate the role of Cx3cl1/Cx3cr1 neuronal-glial cross talk in prion disease we inoculated female *Cx3cr1*^*-/-*^ mice and Balb/c wild type controls intracerebrally with three different mouse-adapted prion strains. Chandler/RML and ME7 are two distinct scrapie-derived prion strains and MRC2 was generated from the passage of cattle BSE in mice [19,20]. Each of these prion strains transmits readily to wild type mice and produce a distinctive incubation time and patterns of PrP^Sc^ distribution, gliosis and spongiosis [19]. Onset of diagnostic signs of prion disease was significantly earlier in *Cx3cr1*^*-/-*^ mice relative to controls for all three prion strains (Figure 1). For Chandler/RML prions the mean incubation time (days ± sem) was reduced by 4% from 150 ± 2 in *Cx3cr1*^*+/+*^ to 144 ± 1 in *Cx3cr1*^*-/-*^ mice (P = 0.004, Kaplan-Meier log rank survival) and for ME7 the mean incubation time was reduced by 7% from 178 ± 1 in *Cx3cr1*^*+/+*^ to 168 ± 1 in *Cx3cr1*^*-/-*^ mice (P < 0.0001, Kaplan-Meier log rank survival). For MRC2 the mean incubation time was reduced by 7% from 206 ± 2 in *Cx3cr1*^*+/+*^ to 200 ± 1 in *Cx3cr1*^*-/-*^ mice (P < 0.0001, Kaplan-Meier log rank survival).

### Neuropathology

**Figure 2:**
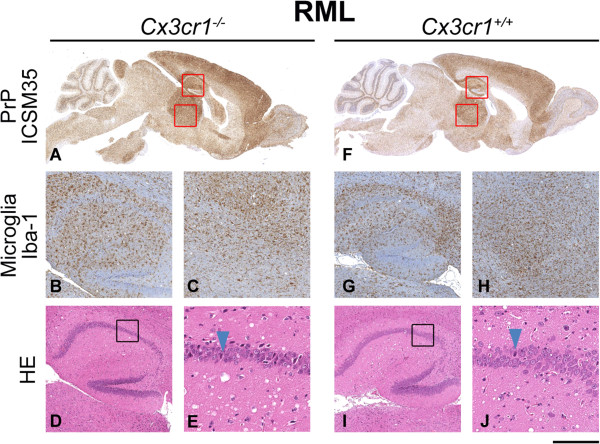
**Chandler/RML neuropathology.** Histological features of Chandler/RML prion transmission to *Cx3cr1*^*-/-*^**(A-E)** and wild type control **(F-J)** mice. Panels **A** and **F** show the global distribution of disease-associated PrP using anti-PrP monoclonal antibody ICSM35. Panels **B**, **C**, **G** and **H** show staining of microglia with Iba-1 in the hippocampus **(B, G)** and the thalamus **(C, H)**. Panels **D**, **E**, **I** and **J** are stained with haematoxylin and eosin (H&E) to visualise spongiosis and neuronal loss in the hippocampus. Boxes show where the higher power images have been taken and arrows highlight apoptotic cells. Overall, no differences are seen between *Cx3cr1*^*-/-*^ and wild type mice. Scale bar corresponds to 2 mm **(A, F)**, 400 μm **(B, C, D, G, H, I)** or 100 μm **(E, J)**.

**Figure 3:**
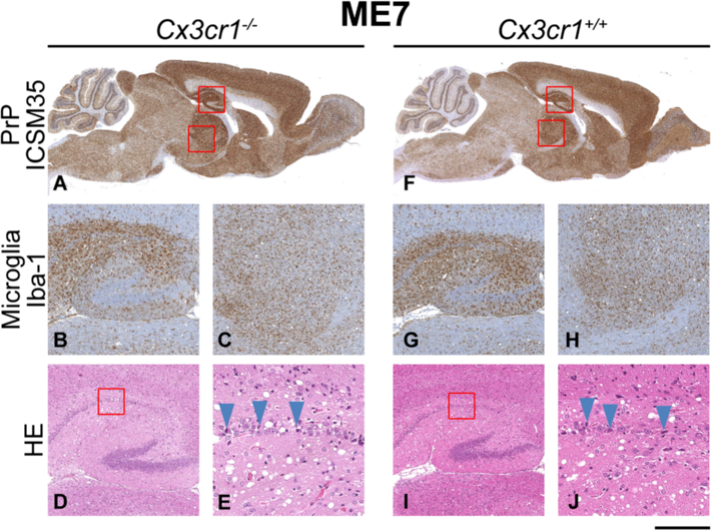
**ME7 neuropathology.** Histological features of ME7 prion transmission to *Cx3cr1*^*-/-*^**(A-E)** and wild type control **(F-J)** mice. Panels **A** and **F** show the global distribution of disease-associated PrP using anti-PrP monoclonal antibody ICSM35. Panels **B**, **C**, **G** and **H** show staining of microglia with Iba-1 in the hippocampus **(B, G)** and the thalamus **(C, H)**. Panels **D**, **E**, **I** and **J** are stained with haematoxylin and eosin (H&E) to visualise spongiosis and neuronal loss in the hippocampus. Boxes show where the higher power images have been taken and arrows highlight apoptotic cells. Overall, no differences are seen between *Cx3cr1*^*-/-*^ and wild type mice. Scale bar corresponds to 2 mm **(A, F)**, 400 μm **(B, C, D, G, H, I)** or 100 μm **(E, J)**.

**Figure 4:**
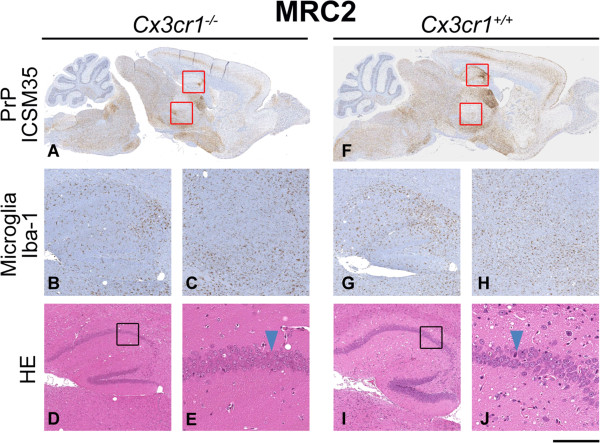
**MRC2 neuropathology.** Histological features of MRC2 prion transmission to *Cx3cr1*^*-/-*^**(A-E)** and wild type control **(F-J)** mice. Panels **A** and **F** show the global distribution of disease-associated PrP using anti-PrP monoclonal antibody ICSM35. Panels **B**, **C**, **G** and **H** show staining of microglia with Iba-1 in the hippocampus **(B, G)** and the thalamus **(C, H)**. Panels **D**, **E**, **I** and **J** are stained with haematoxylin and eosin (H&E) to visualise spongiosis and neuronal loss in the hippocampus. Boxes show where the higher power images have been taken and arrows highlight apoptotic cells. Overall, no differences are seen between *Cx3cr1*^*-/-*^ and wild type mice. Scale bar corresponds to 2mm **(A, F)**, 400 μm **(B, C, D, G, H, I)** or 100 μm **(E, J)**.

Brain sections from terminally sick mice from each group were evaluated for evidence of neuropathological changes. Overall, for Chandler/RML inoculated mice, the patterns of spongiosis, gliosis and PrP distribution were the same for both wild type and *Cx3cr1*^*-/-*^ groups. Even in the absence of Cx3cl1/Cx3cr1 signalling, microglia show extensive proliferation and based on morphology appear to be activated and similarly distributed to that seen in the controls (Figure 2). Similarly, for ME7 and MRC2 inoculated animals, no differences were seen in the patterns of spongiosis, gliosis, PrP and microglia distribution between *Cx3cr1*^*-/-*^ and *Cx3cr1*^*+/+*^ groups (Figures 3 and 4). Neuropathological differences are seen between the three prion strains, however, these are characteristic of the strains and do not appear to have been influenced by loss of Cx3cr1.

### Prion strains

Prion strains in mice are characterised by their incubation time, neuropathology and the biochemical properties of PrP^Sc^[13]. To evaluate the effect of Cx3cr1 knockout on the faithful maintenance of strain characteristics we examined the brains of infected animals by western blotting using the anti-PrP antibody ICSM35. No differences were seen in PrP^Sc^ type between *Cx3cr1*^*-/-*^ and *Cx3cr1*^*+/+*^ animals (Additional file 1 and Additional file 2: Figure S1).

### Cytokine mRNA expression

**Figure 5:**
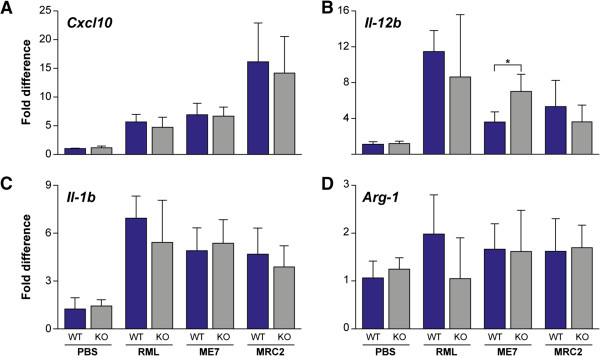
**Chemokine/cytokine mRNA expression.** Quantification of chemokine/cytokine mRNA expression from half mouse brain by real-time RT-PCR. N = 5 for all groups (except uninfected control, wild type mice where n = 4) and samples were run in triplicate. All samples were duplexed for the chemokine/cytokine (Fam-label) and an endogenous control *GAPDH* or *β-actin* (Vic-label). Expression level is shown as the fold difference between the wild type uninfected control and all other groups (*y*-axis). Error bars represent the standard deviation. **(A)***Cxcl10*, **(B)***Il-12b*, **(C)***Il-1b*, **(D)***Arg-1*. WT = wild type *Cx3cr1*^*+/+*^, KO = Cx3cr1^-/-^. *P = 0.01 (*T*-test). For **A** (P < 0.01), **B** (P < 0.05) and **C** (P < 0.01) expression levels for all infected samples are significantly increased relative to the uninfected controls. No statistically significant differences were seen for **D**.

Based on immunohistochemical staining with Iba1, we saw no differences in microglial proliferation and distribution between *Cx3cr1* knockout and wild type mice. The activation status of microglia may be inferred by the relative abundance of various cytokines and chemokines in the brain. In other models of neurodegeneration in the absence of Cx3cr1, alterations in the cytokine/chemokine environment has been observed [8,11]. We therefore measured the mRNA levels, by real time RT-PCR, of various cytokines/chemokines in the brains of terminally sick mice for each of our three prion strains and in uninfected controls for both wild type and *Cx3cr1* knockout mice. Firstly, we looked at levels of cytokines/chemokines known to be induced in prion disease (*Cxcl10*, Il-*12b* and *Il-1b*) [17]. *Cxcl10* expression was significantly increased in all three prion strains relative to uninfected controls with 5-fold increases for Chandler/RML and ME7 and a 15-fold increase for MRC2 (P < 0.01 for all prion strains, *T*-test). No significant differences were seen between knockout and wild type groups (Figure 5A). *Il-12b* expression was also significantly increased in all prion-infected groups (P < 0.05 for all prion strains). However, a 2-fold increase was seen in *Cx3cr1*^*-/-*^ relative to *Cx3cr1*^*+/+*^ mice (P = 0.01) but only with the ME7 prion strain (Figure 5B). *Il-1b* expression was significantly increased in all prion-infected groups (P < 0.01 for all strains, *T*-test), however, no significant differences were seen between *Cx3cr1*^*-/-*^ and wild type controls (Figure 5C). Microglia may be activated through either a classical (M1) or alternative (M2) pathway [24]. Cxcl10 is a marker of the M1 pathway and Arg-1 is a marker of the M2 pathway. As described, *Cxcl10* mRNA expression is induced equally in both Cx3cr1 knockout and wild type mice with all prion strains, however, no differences were observed for *Arg-1* mRNA expression by mouse genotype or prion infection (Figure 5D). This suggests that the M1 microglia activation pathway is triggered by prion infection and this is not altered by loss of Cx3cr1. We also looked at *Cx3cl1* (fractalkine) mRNA expression and saw no significant differences between *Cx3cr1* knockout and wild type mice or between infected and uninfected groups (Additional file 1 and Additional file 3: Figure S2).

## Discussion

We have shown that Cx3cr1 deficiency results in an acceleration in the onset of the clinical signs of prion disease, as illustrated by shorter incubation times, with three different prion strains. These data suggest that in wild type animals intact Cx3cl1/Cx3cr1 signalling is partially protective and may act to somewhat restrain the microglial response. The difference in incubation time observed in knockout mice is quantitatively modest but highly statistically significant with a reduction of 4% for Chandler/RML and 7% for both ME7 and MRC2 prion strains and no overall differences observed in microglial activation either by neuropathology or cytokine/chemokine expression in terminally sick mice. These data suggest that Cx3cl1/Cx3cr1 signalling plays only a minor role in modulating microglial activation in prion disease.

Although microglial activation was unaltered in our study it is worth noting that for both neuropathological examination and cytokine/chemokine production, we looked only at brains from terminally sick animals. It is possible that microglia are activated earlier in the absence of Cx3cr1 but by end stage disease microglia are activated and distributed as seen in wild type mice. If microglia are activated earlier in *Cx3cr1*^*-/-*^ mice the earlier exposure to neurotoxic factors may contribute to the earlier onset of clinical disease. We observed a 2-fold increase in *Il-12b* expression in *Cx3cr1*^*-/-*^ relative to *Cx3cr1*^*+/+*^ mice (P = 0.01) but only with the ME7 prion strain which suggests a prion strain specific effect. Given that this was not observed with other proinflammatory cytokines or with the other prion strains it is difficult to assess the relevance of this finding.

The role of Cx3cl1/Cx3cr1 signalling in neurodegeneration is variable, highly dependent on the model used and the phenotype measured and probably reflects the nature of the neuroinflammatory response triggered by each disease process. Our data suggest that for prion disease the neuronal-microglia cross talk provided by this interaction is partially protective but is not sufficient to override other pathways of microglial activation or neuronal loss. The protective role of Cx3cl1/Cx3cr1 is also seen in models of AD (APPPS1 and R1.40), ALS (Tg*SOD1*^G93A^), tauopathy and PD [8-11]. This suggests that different neurodegenerative diseases share common pathways that ultimately contribute to neuronal death and that the relative importance of each pathway is governed by the nature of the original insult.

## Conclusion

We have demonstrated that loss of Cx3cr1 results in an acceleration of onset of prion disease with three distinct prion strains (Chandler/RML, ME7 and MRC2) suggesting that intact Cx3cl1/Cx3cr1 signalling is partially protective in prion disease. This occurs in the context of unchanged microglial activation, as assessed by cellular distribution and cytokine profiles. The role of Cx3cl1/Cx3cr1 signalling in different models of neurodegeneration is highly variable suggesting that its influence is dependent on the precise cytokine environment created by each disease process.

## Abbreviations

AD: Alzheimer’s disease; ALS: Amyotrophic lateral sclerosis; BFS: Buffered formal saline; BSE: Bovine spongiform encephalopathy; CJD: Creuzfeldt-Jakob disease; CNS: Central nervous system; D-PBS: Dulbecco’s phosphate buffered saline; GFAP: Glial fibrillary acid protein; GFP: Green fluorescent protein; H&E: Haematoxylin and eosin; PD: Parkinson’s disease; PrPc: Normal cellular prion protein; PrPSc: Abnormal disease associated isoform of prion protein; RT-PCR: Reverse transcriptase-polymerase chain reaction; TSE: Transmissible spongiform encephalopathy.

## Competing interests

JC is a director and shareholder of D-Gen Limited, an academic spin-out company in the field of prion diagnosis, decontamination and therapeutics. D-Gen markets the monoclonal antibody ICSM35 used in this study.

## Authors’ contributions

SL planned the experiments; SA, GJ, and SL carried out the experimental work. SB and SL analysed the data; SL and JC wrote the manuscript. All authors read and approved the final manuscript.

## Supplementary Material

Additional file 1 Methods-western blotting and additional figure legends. Click here for file

Additional file 2: Figure S1 Western blots of PrP^Sc^ from infected mouse brains. Click here for file

Additional file 3: Figure S2 Cx3cl1 mRNA expression. Click here for file
