# Supplementary material for: Microglial Cx3cr1 knockout reduces prion disease incubation time in mice
Source: BMC Neurosci. 2014 Mar 21;15:44. doi: 10.1186/1471-2202-15-44 (PMC3998043; doi:10.1186/1471-2202-15-44)
Supplement: Additional file 1 — Methods-western blotting and additional figure legends. [file 1471-2202-15-44-S1.docx]

**Additional file 1**

**Methods - western blotting**

10% (weight/volume) brain homogenates in D-PBS were prepared by ribolysing half brains from mice diagnosed with prion disease. Samples were benzonase treated, proteinase K digested (50µg/ml of proteinase K for 1h at 37^o^C) and blotted as described previously (Wadsworth et al., 2001). Anti-PrP monoclonal antibody ICSM35 (D-Gen Ltd, UK) (Asante E et al., 2002) and alkaline-phosphatase-conjugated anti-mouse IgG secondary antibody (Sigma-Aldrich) developed in the chemiluminescent substrate CDP-Star (Tropix Inc) was used for the detection of PrP^Sc^.

**Figure S1 Western blots of PrP^Sc^ from infected mouse brains**

10% w/v brain homogenates (n=3 per group) were digested with proteinase K and immunoblotted with anti-PrP monoclonal antibody ICSM35 (D-Gen Ltd, UK). (A) Transmission of Chandler/RML prions to *Cx3cr1^-/-^* (KO) and *Cx3cr1^+/+^* (WT) controls. (B) Transmission of ME7 prion strain to *Cx3cr1^-/-^* and *Cx3cr1^+/+^* controls. (C) Transmission of MRC2 mouse adapted BSE prion strain to *Cx3cr1^-/-^* and *Cx3cr1^+/+^* controls. No differences were seen between the two groups regardless of prion strain.

**Figure S2 Cx3cl1 mRNA expression**

Quantification of *Cx3cl1* mRNA expression from half a mouse brain by real-time RT-PCR. N=5 for all groups (except uninfected control, wild type mice where n=4) and samples were run in triplicate. All samples were duplexed for the chemokine/cytokine (Fam-label) and an endogenous control *GAPDH* or *β-actin* (Vic-label). Expression level is shown as the fold difference between the wild type uninfected control and all other groups (*y*-axis). Error bars represent the standard deviation. WT = wild type *Cx3cr1^+/+^*, KO = Cx3cr1^-/-^. No significant differences were seen (T-test).
