# Supplementary figures and images for: Microglial Cx3cr1 knockout reduces prion disease incubation time in mice
Source: BMC Neurosci. 2014 Mar 21;15:44. doi: 10.1186/1471-2202-15-44 (PMC3998043; doi:10.1186/1471-2202-15-44)

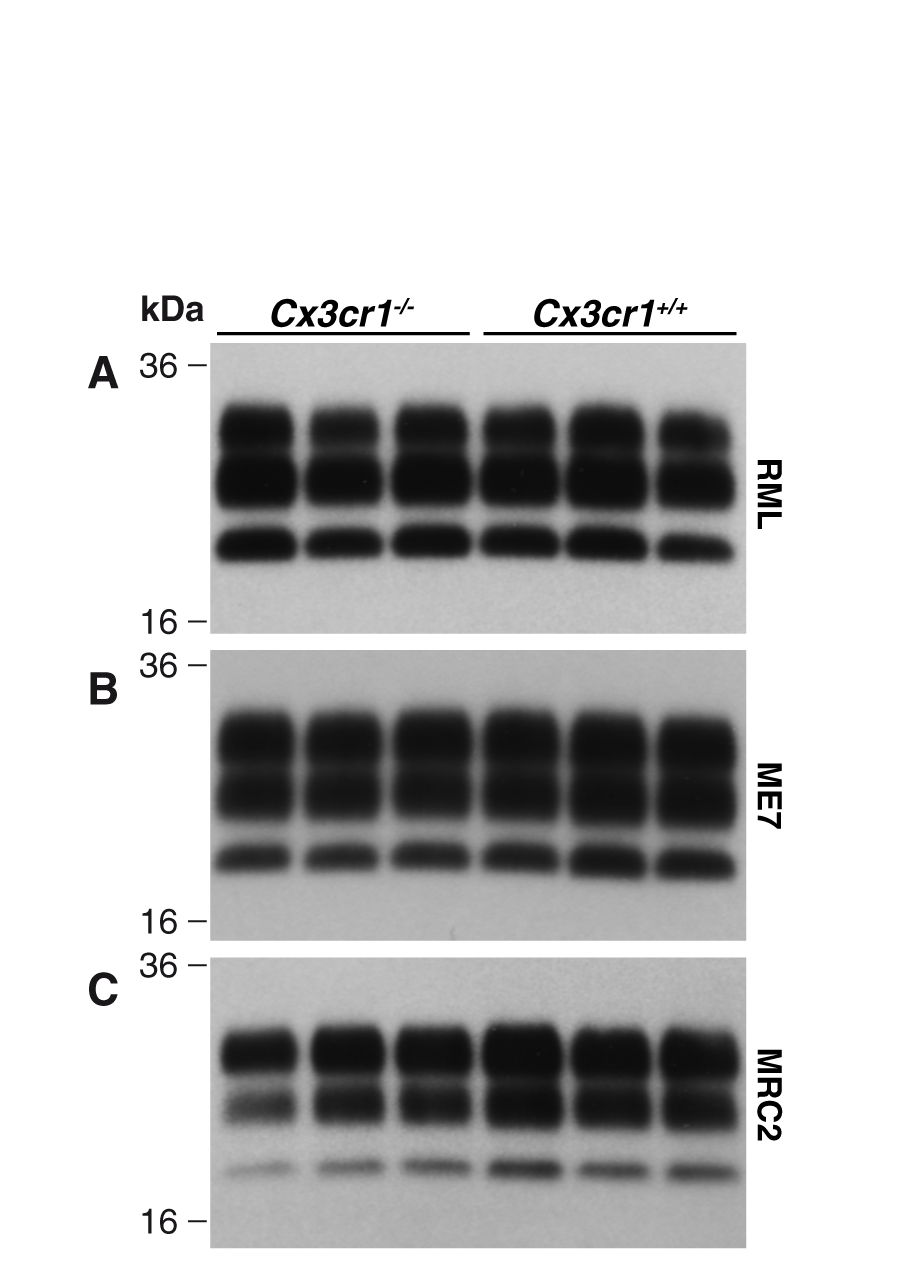

Supplement: Additional file 2: Figure S1 — Western blots of PrPSc from infected mouse brains. [file 1471-2202-15-44-S2.tiff]

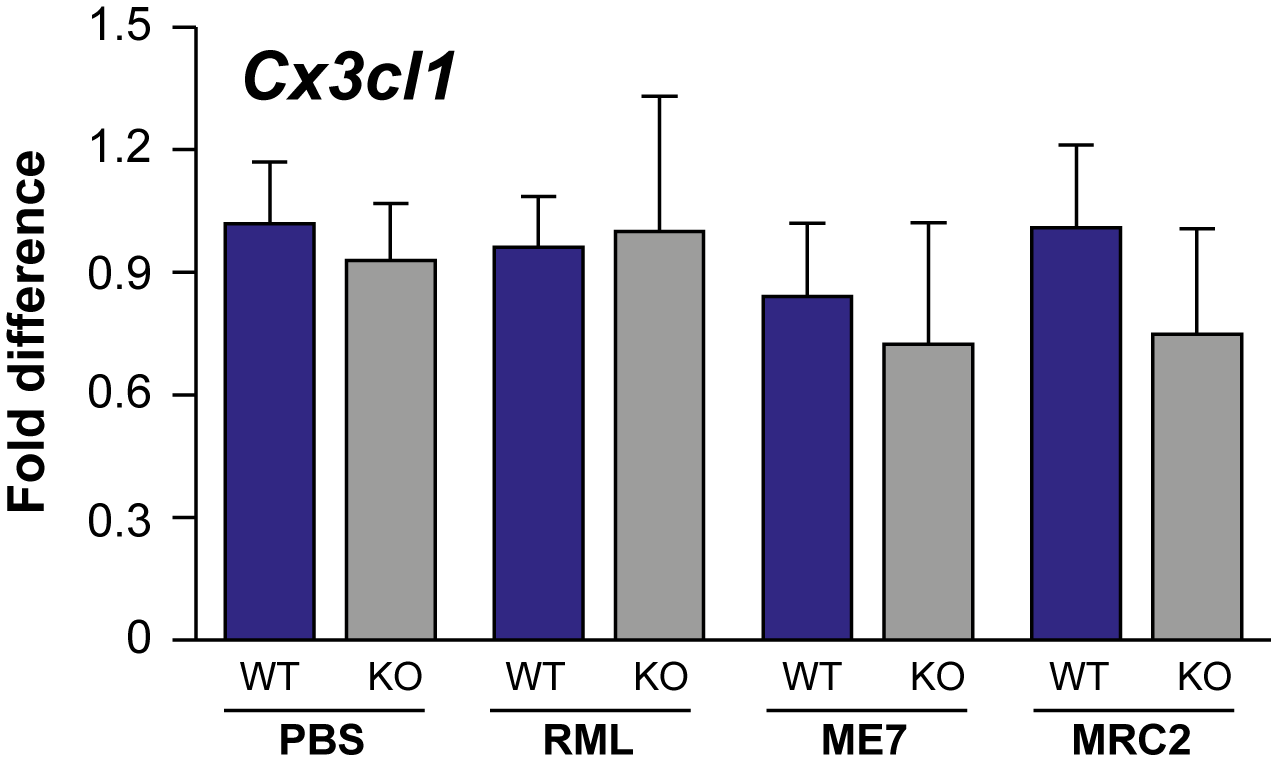

Supplement: Additional file 3: Figure S2 — Cx3cl1 mRNA expression. [file 1471-2202-15-44-S3.tiff]
